# Comparison of different extraction techniques to profile microRNAs from human sera and peripheral blood mononuclear cells

**DOI:** 10.1186/1471-2164-15-395

**Published:** 2014-05-23

**Authors:** Marjorie Monleau, Sophie Bonnel, Thierry Gostan, Dominique Blanchard, Valérie Courgnaud, Charles-Henri Lecellier

**Affiliations:** Institut de Génétique Moléculaire de Montpellier UMR 5535 CNRS, 34293 Montpellier cedex 5, France; Université Montpellier 2, Place Eugène Bataillon, 34095 Montpellier cedex 5, France; Université Montpellier 1, 5 Bd Henry IV, 34967 Montpellier cedex 2, France; Prestizia/Theradiag group, Cap Alpha, 9 avenue de l’Europe, F-34830 Clapiers, France

## Abstract

**Background:**

microRNAs (miRNAs) play crucial roles in major biological processes and their deregulations are often associated with human malignancies. As such, they represent appealing candidates as targets of innovative therapies. Another interesting aspect of their biology is that they are present in various biological fluids where, advantageously, they appear to be very stable. A plethora of studies have now reported their potential as biomarkers that can be used in diagnosis, prognosis and/or theranostic issues. However, the application of circulating miRNAs in clinical practices still requires the identification of highly efficient, robust and reproducible methods for their isolation from biological samples.

In that context, we performed an independent cross-comparison of three commercially available RNA extraction kits for miRNAs isolation from human blood samples (Qiagen and Norgen kits as well as the new NucleoSpin miRNAs Plasma kit from Macherey-Nagel). miRNAs were further profiled using the Taqman Low Density Array technology.

**Results:**

We found that, although these 3 kits had equal performances in extracting miRNAs from peripheral blood mononuclear cells, the Macherey-Nagel kit presented several advantages when isolating miRNAs from sera. Besides, our results have indicated that, depending on the quantity of the biological samples used, the extraction procedure directly impacted on the G/C composition of the miRNAs detected.

**Conclusion:**

Overall, our study contributes to the definition of a reliable framework for profiling circulating miRNAs.

**Electronic supplementary material:**

The online version of this article (doi:10.1186/1471-2164-15-395) contains supplementary material, which is available to authorized users.

## Background

MicroRNAs (miRNAs) are a class of small noncoding RNAs (typically 20–23 nt) that are important regulators of gene expression at the post-transcriptional level. In recent years, numerous studies have involved miRNA disregulations in various diseases and the number of miRNA publications is growing each year. To date, more than a thousand miRNAs have been identified and their presence in various body fluids (plasma, serum, urine…) as well as their remarkable stability make them excellent candidates for non-invasive biomarkers of various human diseases [1]. For the development of miRNAs-based biomarkers, several issues associated with samples manipulation, miRNAs extraction, measurements and statistics need to be addressed [2–4]. For instance, several studies have shown the importance of samples processing [5, 6]. Likewise, it was reported that hemolysis occurring during blood collection has significant impact on the miRNAs content in plasma/serum [7–10]. The evaluation of the quantity and quality of miRNAs isolated from biological samples is indeed a key step in miRNA profiling studies. Although methods for miRNA extraction are usually similar to that used in the case of total RNAs (with only slight modifications required to retain the small RNA fraction), the sizes and relative abundance of ribosomal RNAs cannot give information about the integrity of the miRNA preparation. In addition, the quantification of miRNA preparations can only be accurate in samples where larger RNAs are not degraded as the degradation products can compromise this quantification. Moreover, the low concentration of RNAs present in body fluids makes the estimation of miRNAs abundance particularly difficult [11]. Another aspect that can impact miRNA profiling is the qPCR efficiency that can be affected by minute amounts of inhibiting compounds co-extracted with RNA [12]. Besides, it has been reported that short RNAs with low GC content may be selectively lost during extraction from a small number of cells, depending on the extraction methods [13]. It is thus crucial to compare different protocols in order to identify the most reproducible and reliable method. Several studies have indeed tackled this point, revealing different performances between the commercially available kits for the isolation of miRNAs [12, 14–17]. Here, we broadened these analyses and compared, using Taqman Low Density Arrays, miRNAs profiles of peripheral blood mononuclear cells (PBMCs) and sera from human healthy donors, obtained with three distinct commercial kits: Qiagen, Norgen Biotek Corporation and Macherey-Nagel. To the best of our knowledge the Macherey-Nagel kit for plasma/serum has not been studied before. In this paper, we showed that the quantity and the quality of RNAs extracted from PBMCs with these three kits did not significantly differ. This was in contrast to miRNAs extraction from serum for which the Macherey-Nagel kit presented several advantages. Besides, we found that, irrespective of the kit used, increasing the quantity of the starting biological materials (PBMC or serum) introduced a bias in the isolation of miRNAs and favored the extraction of G/C low miRNAs. We also bring evidence that the optimal detection of miRNAs is not necessarily obtained with the maximum quantity of total RNAs.

## Methods

### Samples isolation

Blood samples were obtained from ten blood donors who underwent a brief medical examination (*Etablissement Français du Sang* (EFS) Montpellier, France). These blood samples were obtained in accordance to the ethical guidelines of the French Ministry of Health (Code de Santé Publique Article L1131-1 and next). This study was approved by the ethics committee of the EFS-Pyrénées-Méditerranée (EFS-PM- Agreement: # 21/PLER/MTP/CNR02/2013-007). All donors have given their written consent for non-therapeutic use of their blood sample donation. Whole blood samples from each donors were collected in two Vacutainer tubes, one being EDTA coated and the other non-EDTA coated. RNase-free protocols were followed throughout all procedures. Sera were prepared from blood collection tubes without anticoagulant after centrifugation at 500 g for 10 minutes at room temperature and inspected visually for any pink hue, which is indicative of hemolysis [8], and then, immediately frozen at −80°C. PBMCs were isolated, from EDTA tubes, by Ficoll density gradient centrifugation (Sigma-Aldrich). 3×10^6^ or 1×10^6^ cells were mixed with lysis buffer according to the manufacturers’ instructions for each RNA extraction kit, in order to achieve lysis and inactivate endogenous RNAses. Lysates from PBMCs were frozen in the lysis buffer at −80°C until next steps of RNA purification. Experiments were performed with RNAs thawed only once.

### RNA extraction from PBMCs and serum samples

Total RNA was extracted from the PBMCs lysates (1×10^6^ or 3×10^6^ cells) using the miRNeasy mini kit (reference 217004, Qiagen, CA), the Total RNA Purification Kit (product 17200, Norgen Biotek Corporation, Canada) and the NucleoSpin miRNAs kit (reference 740971, Macherey-Nagel, Düren, Germany) following the manufacturers’ protocols. Qiagen and Norgen kits require a Phenol/Chloroform extraction step unlike the kits from Macherey-Nagel. The Macherey-Nagel protocol allows isolating both small and large RNAs in one or two fractions. Here, we have chosen to extract total RNAs in one fraction. Total RNAs were eluted in 30 μL nuclease-free water for Qiagen kit or 50 μL for Norgen and for Macherey Nagel kits.

Total RNAs were extracted from thawed serum samples using the miRNeasy mini kit (Qiagen, CA), the Plasma/Serum Circulating RNA purification Kit (product 30000, Norgen Biotek Corporation, Canada) and the NucleoSpin miRNAs Plasma kit (reference 740981, Macherey-Nagel, Düren, Germany). For both Qiagen and Macherey Nagel kits, total RNAs were extracted from 300 μL or 600 μL of serum and further eluted in 30 μL of nuclease-free water. For the Norgen kit, total RNAs were extracted from 200 μL of serum and eluted in 50 μL of nuclease-free water.

### RNA quality

Total RNA concentration was expressed as micrograms or nanograms RNA per million cells (for PBMCs) or per milliliter of serum. The RNA concentration and quality were first assessed using the NanoDrop ND-1000 spectrophotometer (NanoDrop Technologies, DE). PBMC sample purity was estimated by measuring the ratio of spectrophotometric absorbance (260 nm/280 nm). For a pure RNA sample, this ratio should be comprised between 1.8 and 2 RNAs were further analyzed with the Bioanalyzer 2100 (Agilent Technologies, CA) using small RNA and RNA 6000 Nano chips (Agilent, CA). The RNA Integrity Number (RIN) obtained by the Nano 6000 kit for PBMCs indicates the RNA quality of a sample (RIN values >8 are commonly considered as high-quality RNA). Small RNA (from 6 to 150 nt) and miRNAs (from 6 to 40 nt) concentrations as well [micro/small] RNA ratio were calculated from the electropherogram of the Small RNA kit for PBMC and sera.

### TaqMan low-density arrays (TLDA) for miRNAs profiling

MicroRNA profiling of samples was performed using TaqMan Array Human MicroRNA panels A and B (Life technologies, CA). Each TLDA card detects 384 features including 377 human miRNAs, three endogenous small RNA controls (one of them being in quadruplicate), and a negative control. 754 human miRNAs were quantified in total. Reverse transcription and pre-amplification were performed following the manufacturer’s instructions (Life technologies, CA). Briefly, 3 μL RNAs from serum or 10 to 300 ng RNAs from PBMCs were reverse transcribed using the Megaplex reverse transcription (RT) reaction in a final volume of 7.5 μL using stem-loop primers designed by Life Technologies. 2.5 μL of this cDNA solution were used for a pre-amplification step in a final volume of 25 μL then diluted four times in distilled water DNase/RNase free (Life Technologies Gibco). Nine μL of diluted pre-amplified product added to 900 μL of total mix were used per TLDA card. Real time quantitative PCR was performed with ViiA7 real-time PCR system, and data were collected with the manufacturer’s ViiA™ Software. Gene Expression Suite software (Applied Biosystems, CA) was further used to process the array data. Automatic thresholds were checked individually and corrected when necessary.

### Data analysis and statistical methods

Data processing and analysis were conducted using tools from Microsoft Excel and Prism GraphPad V5.0d software (GraphPad Software, CA). Comparisons of quantity and quality data were performed using Mann Whitney or Kruskal-Wallis test depending on the distribution of the data. Only miRNAs with a cut-off of cycle threshold (Ct) < 32 were considered in PBMCs while no cut-off was applied in the case of serum since in this fluid, the quantities of starting materials are much lower. Correlations were calculated using the Spearman method. For the Bland-Altman analysis, the association between the difference and the average was evaluated by the coefficient of correlation and tested by the non-zero correlation test. Wilcoxon test was used to assess whether the median difference (bias) between the two conditions was significantly different from 0 and the limits of agreement were defined as median difference +/−1.96 SD. The thermodynamic stability of miRNAs (kcal/mol) was calculated using Quikfold from the DINAMelt web server (http://mfold.rna.albany.edu/?q=DINAMelt/Quickfold) [18].

## Results

### Reproducibility of miRNAs expression profiles using TLDA cards

**Figure 1:**
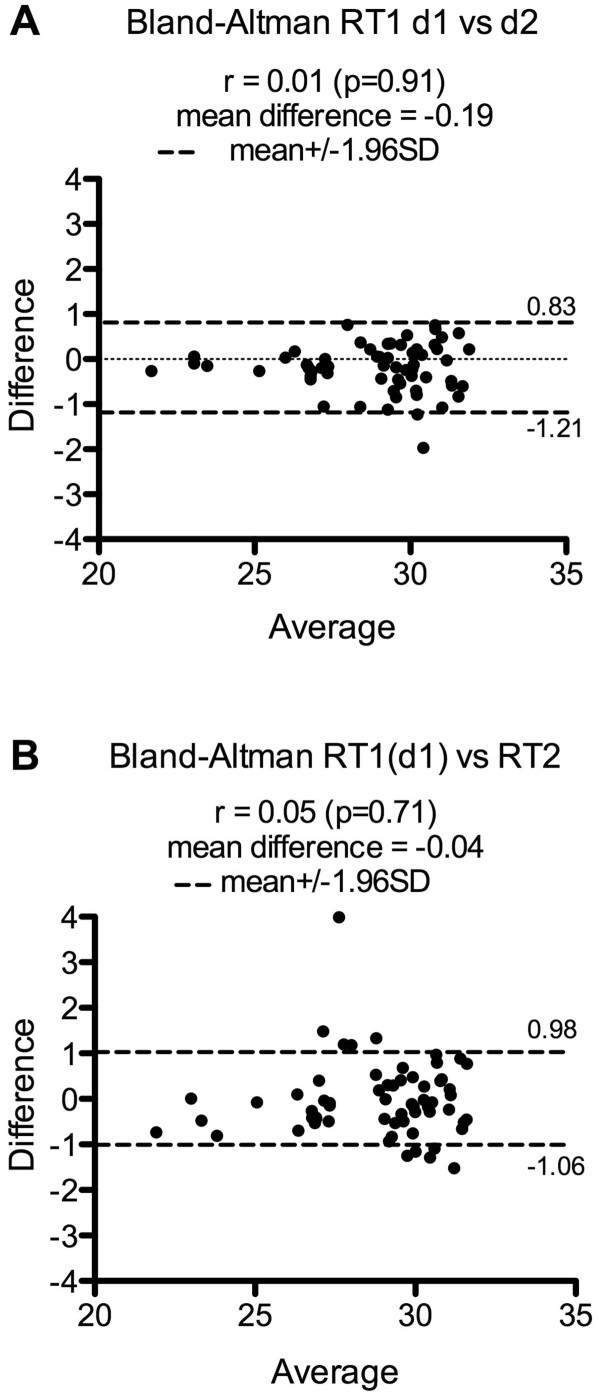
**Assessment of the TLDA reproducibility.** The TLDA reproducibility was assessed by testing, from one RNA sample, two separate qPCR amplifications from **A**- the same RT and pre-amplification product (RT1-d1 vs RT1-d2: Bland-Altman) and **B**- separate RT and pre-amplification reactions (RT1 (d1) vs RT2: Bland-Altman). RT with 150 ng of RNA isolated from 3×10^6^ cells with Macherey-Nagel kit. Only miRNAs with Ct < 32 were considered.

The reproducibility of the miRNA expression profiling using Taqman Low Density Array was first assessed by comparing two separate qPCR experiments using the same RT product obtained from total RNA isolated from PBMCs with the recently available Macherey-Nagel (MN) extraction kit. Seventy-one miRNAs were detected in the first experiment (d1) and 79 in the second (d2), with 63 miRNAs detected and 670 miRNAs not detected in both experiments (i.e. 96.8% of reproducibility). The correlation of the Ct values of miRNAs commonly detected was high (r = 0.93) and a Bland-Altman plot showed small mean difference (−0.19) with only one miRNA outside the limits of agreement (Figure 1A). Secondly, two RT replicates from the same RNA sample (150 ng) were compared. RT1 (d1) allowed detecting 71 miRNAs while RT2 detected 89 miRNAs. 63 miRNAs were detected in common while 663 miRNAs were undetectable (i.e. 95.9% of reproducibility). The correlation coefficient was 0.90 and a Bland-Altman plot displayed small mean difference (−0.04) between Ct values with only five miRNA Ct values outside the limits of agreement (Figure 1B).

**Figure 2:**
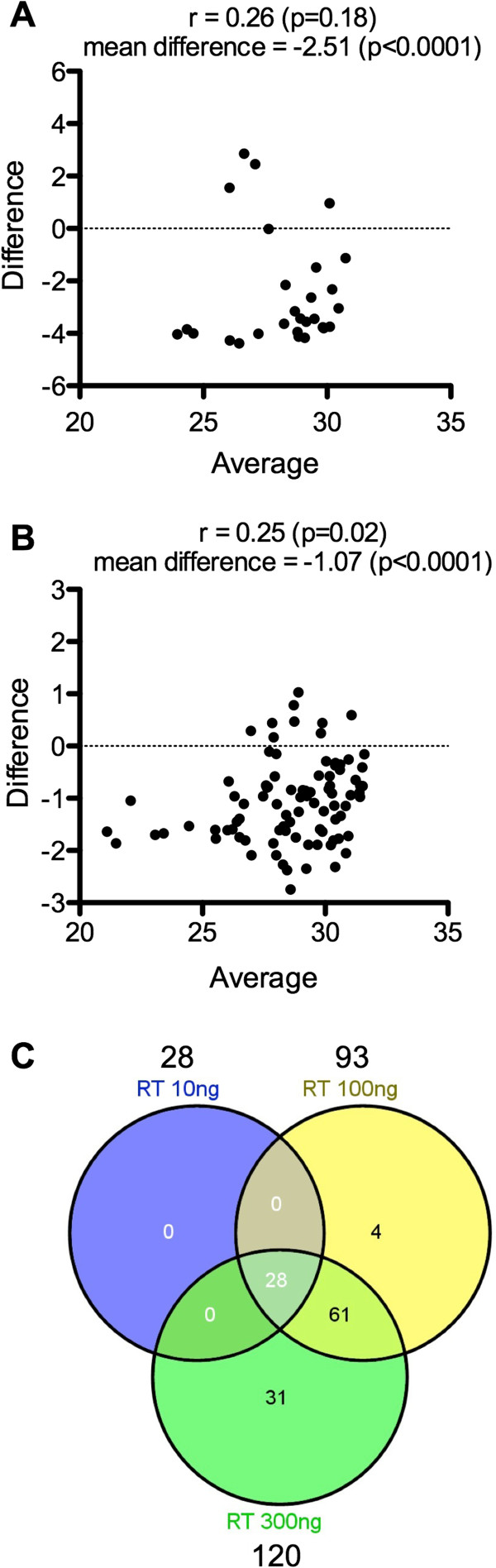
**Impact of the RNA quantity on miRNA TLDA profiles.** miRNA TLDA profiles were compared using three different RNA quantities for RT (10, 100 and 300 ng), of the same RNA sample extracted from 3×10^6^ PBMCs by Macherey-Nagel. **A**- Bland-Altman analysis: condition 100 ng of RNA for RT versus 10 ng. **B**- Bland-Altman analysis: condition 300 ng of RNA for RT versus 100 ng. **C**- Venn diagram with the detectable miRNAs. Only miRNAs with Ct < 32 were considered. The Pearson correlation coefficient is indicated.

The impact of increasing the amount of total RNAs used for RT in TLDA miRNA profiles was also evaluated. Three RTs were then performed using as starting material of 10, 100 and 300 ng of total RNAs purified from 3×10^6^ PBMCs with the MN kit (Figure 2). As expected, the Ct values of the miRNAs detected in all 3 settings decreased when the RNA quantity used for RT increased: mean Ct difference of −2.51 between 10 and 100 and −1,07 between 100 and 300 (Figure 2A and B). An increase in the number of detectable miRNAs was also logically observed although not perfectly linear (28/93/120 miRNAs using 10, 100 and 300 ng of total RNA, respectively, Figure 2C). The miRNAs detected with 10 ng of total RNAs were detected with 100 ng and miRNAs detected with 100 ng of total RNAs were also detected with 300 ng (Figure 2C). Thus, the detection of miRNAs using the TLDA platform is mostly influenced by the quantity of total RNAs used for RT, not by the technology itself. Together our results showed that TLDA is a reproducible method that is therefore suitable to compare different RNA extraction procedures.

### Comparison of RNA quality and quantity

Macherey-Nagel, Qiagen, and Norgen extraction kits were used to isolate total RNAs (including small and miRNAs) from PBMCs (1×10^6^ cells) and sera (300 μL for Qiagen and MN or 200 μL for Norgen) from 10 healthy donors (Table 1 and Additional file 1: Figure S1). First, we observed that the quantity of RNA isolated from PBMCs with the Norgen kit measured by Nanodrop (1.93 μg/1×10^6^ cells, Table 1A) was higher than that obtained with MN (p = 0.006 – Mann Whitney test) or Qiagen (p = 0.002 – Mann Whitney test) while quantity measurements with Agilent Nano 6000 chips yielded similar results. At the quality level, ratio of absorbance at 260 nm and 280 nm (A260/280) showed that the RNA purity obtained with the MN kit was slightly better than that obtained with Qiagen or Norgen kits (Table 1A, p = 0.0004 and p = 0.0025, respectively - Mann Whitney test). The RINs only slightly differ but higher variability was observed with the samples extracted using the Norgen kit (sd = 2.9). Agilent Small RNA chips did not indicate significant difference in the quantities of small RNA and miRNAs (Table 1A, micro/small RNA ratios of 14.4 (sd = 7.7), 13.6 (1.7) and 15.6 (19.4)% using MN, Qiagen and Norgen extraction kits, respectively).

**Table 1:** **Assessment of quantity and quality of RNA isolated**

A		Macherey Nagel	Qiagen	Norgen
PBMC	n	8	10	10
Nanodrop	Total RNA: Mean quantity (ug/1×10^6^ cells or ml serum) (SD)	1.03 (0.22)	0.97 (0.14)	1.93 (0.69)
	Ratio OD (260 nm/280 nm) (SD)	2.04 (0.07)	1.75 (0.06)	1.91 (0.07)
Agilent Nano 6000 Chip	Total RNA: Mean Quantity (ug/1×10^6^ cells or ml serum) (SD)	1.19 (0.33)	0.96 (0.30)	0.98 (0.50)
	RIN mean (SD)	8.84 (0.34)	8.73 (0.41)	7.33 (2.93)
**B**		**Macherey Nagel**	**Qiagen**	**Norgen**
Serum	n	10	10	10
Nanodrop	Total RNA: Mean quantity (ug/1×10^6^ cells or ml serum) (SD)	0.91 (0.46)	0.89 (0.40)	0.99 (0.34)
	Ratio OD (260 nm/280 nm) (SD)	1.24 (0.11)	1.40 (0.13)	1.27 (0.52)

The quantity and quality of RNA isolated from (A) PBMCs and (B) serum using three RNA extraction kits (Macherey-Nagel, Qiagen, Norgen) were evaluated by Nanodrop, Agilent nano 6000 (PBMCs only) and Agilent small RNA chips.

Second, the quantity and quality of RNAs extracted from the serum samples were analyzed. No significant difference was observed in term of RNA quantities as measured by Nanodrop (Table 1B). Likewise, Agilent small RNA chip analyses did not indicate significant difference in small RNA and miRNAs quantities, although, for some samples, the MN extraction kit yielded higher quantities (Additional file 1: Figure S1 and Table 1B, mean miRNAs concentration of 80, 22 and 11 ng/mL for MN, Qiagen and Norgen extraction protocols, respectively). However the quality of RNA obtained with the MN isolation kit was higher (as indicated by the ratio miRNAs/smallRNA, 61.3%) compared to those obtained with the two other kits (44.5% with Qiagen and 32.9%, p = 0.026 with Norgen-t-test). Again, RIN variability obtained with the Norgen kit was higher than that observed with the two other kits (Table 1, compared sd = 0.52 vs. 0.11 (MN) and 1.4 (Qiagen)). As previously observed [4], the 260 nm/280 nm ratio (<1.8) did not appear as a relevant parameter to assess the miRNA quality in serum. Overall, the MN kit seemed to yield better results compared to the two other kits. However, the Norgen kit presented some inconstancy in term of RNA quality extracted from both PBMCs and sera. We therefore decided to pursue our comparison by TLDA profiling focusing on the MN and Qiagen kits. It is worth mentioning that the MN kit has never been extensively tested.

### Comparison of PBMCs and serum miRNAs profiles

**Figure 3:**
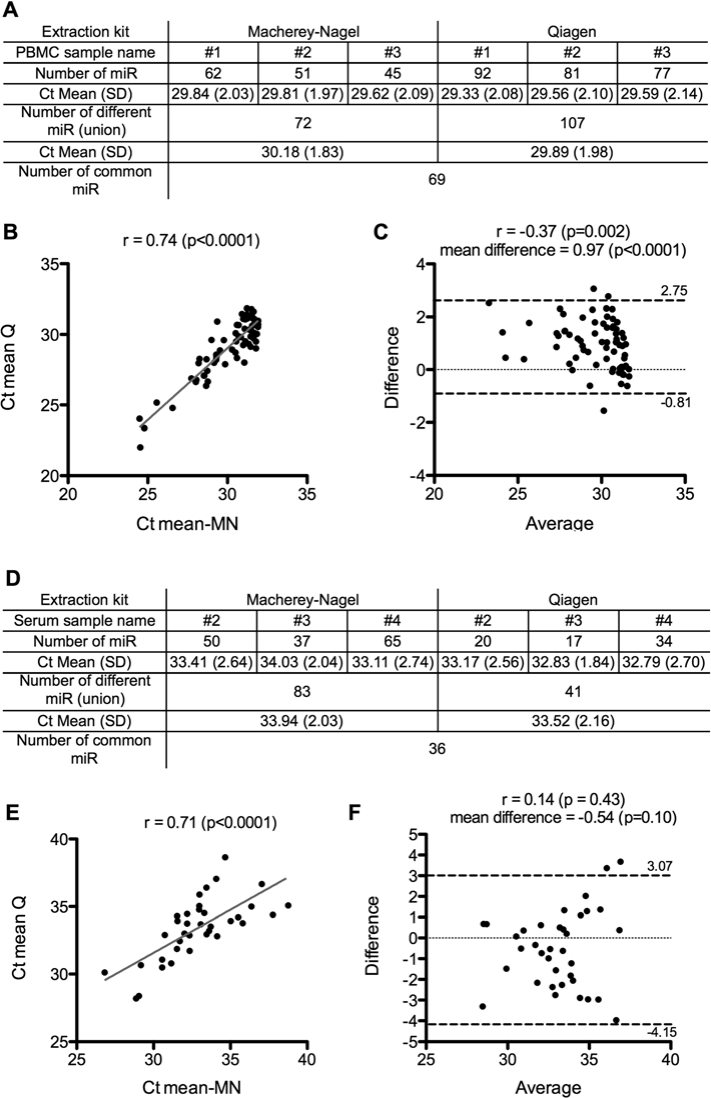
**Comparison of miRNAs expression profiles from human PBMCs and serum.** Human PBMCs (1×10^6^ cells) and serum (300 μL) samples were extracted by Macherey-Nagel (MN) and Qiagen (Q) kits. **A**- Number of miRNAs detected from PBMCs RNA samples using TLDA cards. **B**- Correlation analysis from PBMCs samples. **C**- Bland-Altman analysis MN versus Q from PBMCs. **D**- Number of miRNAs detected from serum RNA samples using TLDA cards. **E**- Correlation analysis from serum samples. **F**- Bland-Altman analysis MN versus Q from serum. TLDA data were obtained from biological triplicate for each extraction kits. Analysis using mean Ct values of triplicate. Only miRNAs with Ct < 32 were considered for PBMCs. No Ct cut-off was applied for serum miRNAs.

Total RNAs were extracted from PBMCs (1×10^6^ cells) and sera (300 μL) of 3 distinct donors using the MN and Qiagen kits. The miRNAs were profiled using a TLDA platform (Figure 3). First, we evaluated the technical and inter-individual variability, for each extraction kits, comparing the profiles of the biological triplicate (Additional file 2: Figure S2). A low variability was observed in PBMC profiles as no significant difference in Ct values (Figure 3A) could be observed for detectable miRNAs (p = 0.89 for MN and p = 0.49 for Qiagen extraction kit, Kruskal-Wallis test) with a Ct mean differences between samples inferior to 1 Ct (Additional file 2: Figure S2A). 72 (9.5%) miRNAs were detected (with Ct < 32) in MN-extracted total RNAs while 107 (14.1%) miRNAs were detected after Qiagen extraction (Figure 3A). 69 miRNAs were detected after extraction with both kits (Figure 3A) with a good correlation of the Ct values (Spearman ρ = 0.74, Figure 3B). The level of expression of miRNAs isolated with Qiagen was slightly higher compared to that observed with MN (Figure 3C), but the Ct mean difference was not greater than 1 Ct. Only three miRNAs had Ct values outside the limits of agreement in a Bland-Altman test (Figure 3C). Thus, for RNA extraction from PBMCs, we concluded that, miRNeasy mini kit from Qiagen and NucleoSpin miRNAs kit from Macherey-Nagel, yielded comparable results.

In the case of serum samples, more variability in the number of miRNAs detected by TLDA for each isolation methods was observed within the biological triplicate (Additional file 2: Figure S2B, Bland-Altman analysis, mean differences ranging from 0.04 to 2.45). However, no significant difference in the Ct values of commonly detected miRNAs (Figure 3D) was observed (p = 0.33 for MN and p = 0.85 for Qiagen extraction kit, Kruskal-Wallis test). 83 miRNAs (11%) were amplified from serum after MN extraction while 41 miRNAs (5.4%) could be detected after Qiagen extraction (Figure 3D). 36 miRNAs were commonly detected with both kits (Figure 3D). Quantities of miRNAs detected in serum samples were very low, with median Ct values around 34 (Figure 3D). Nonetheless, the miRNAs expression profiles evaluated with RNA isolated from both extraction kits correlated (ρ = 0.71, p < 0.0001, Figure 3E). The amounts of miRNAs detected with both kits were similar, with a mean difference of −0.5 (Figure 3F). Only two miRNAs had Ct values outside the limits of agreement of the Bland-Altman test (Figure 3F). Overall, our results indicated that miRNAs profiles from serum samples could be highly variable. These results confirmed that robust statistical tests should be performed when evaluating the potential of circulating miRNAs as diagnostic/prognostic markers [19]. Moreover, we provide evidence that the NucleoSpin miRNAs Plasma kit from Macherey-Nagel is more efficient in extracting miRNAs from serum than the Qiagen miRNeasy mini kit as it allowed detecting twice as many miRNAs.

### Impact of the quantity of the starting biological material on miRNAs profiles

**Figure 4:**
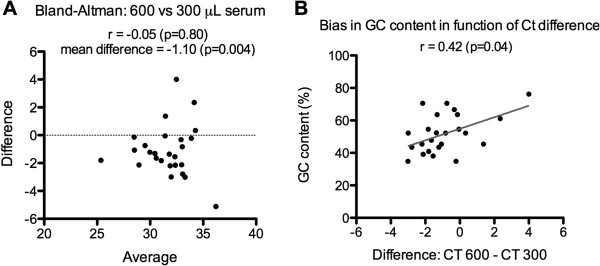
**Assessment of bias in RNA isolation using Macherey-Nagel kit from 300 or 600 μL** **serum. A**- Bland-Altman analysis 600 versus 300 μL of serum. **B**- Plot of the difference in Ct values of the two conditions (x-axis) and the GC content of the miRNAs detected in these two settings (y-axis). The Pearson correlation coefficient is indicated. The GC content corresponds to the percentage of GC in the sequence of mature miRNAs. TLDA datas from biological duplicate. Analysis using mean CT values of common miRNAs, without Ct cut-off.

Then, the performances of the Macherey-Nagel NucleoSpin miRNAs Plasma kit were further examined. First , to evaluate whether increasing the volume of serum could improve miRNAs detection and profiling, the number of detectable miRNAs and their Ct values were compared, in biological duplicate, after RNA extraction from 300 or 600 μL of serum. Nanodrop concentration after elution indicated 13 ng/μL (from 600 μL of serum) and 7 ng/μL (from 300 μL of serum). Using an equal amount of RNA, the total number of detectable miRNAs by TLDA was slightly increased: 57 vs 38 miRNAs and 26 miRNAs were detected with both volumes. A Bland-Altman plot of their Ct values showed that miRNAs extracted from 600 μL of serum were detected with lower Ct values compared to 300 μL with mean difference of −1.10 (Figure 4A). We then computed the correlation between this difference in Ct values and the GC content of the miRNAs (ρ = 0.42, p = 0.04, Figure 4B). Strikingly, this calculation indicated that the sample volumes could impact on the detection of specific miRNAs depending on their G/C composition, in accordance to previous results [13].

**Figure 5:**
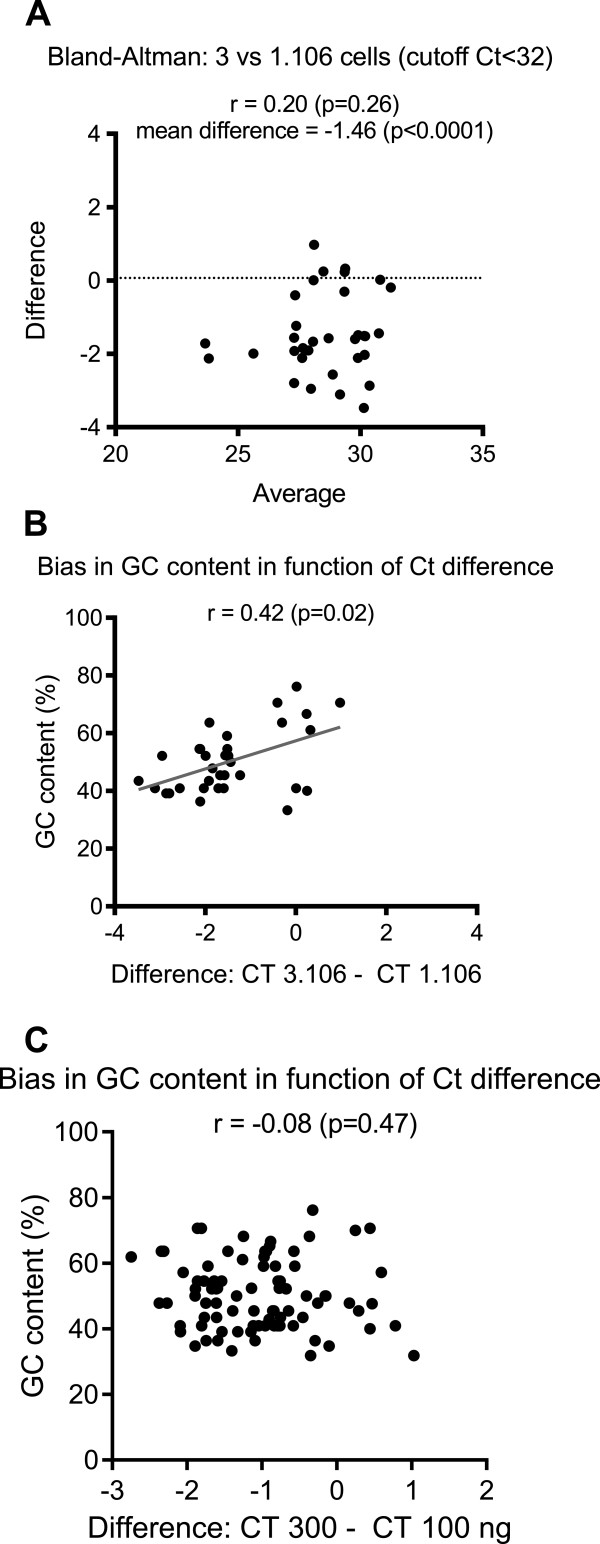
**Assessment of bias in RNA isolation from PBMCs.** Assessment of bias in RNA isolation from PBMCs using the Macherey-Nagel (MN) kit, comparison of extraction from 3×10^6^ and 1×10^6^ cells with a same amount of RNA for RT (130 ng). **A**- Bland-Altman analysis 3×10^6^ versus 1x10^6^ cells. **B**- Plot of the difference in Ct values of the two conditions (x-axis) and the GC content of miRNAs detected in these two settings (y-axis). **C**- Plot of the difference in Ct values of the conditions 300 vs 100 ng (x-axis) and the GC content of the miRNAs detected in these two settings (y-axis). The Pearson correlation coefficient is indicated. TLDA datas from biological duplicate. Analysis using mean CT values of common miRNAs. Only miRNAs with Ct < 32 were considered.

To test if this bias in the GC content was also observed with PMBC-isolated RNAs, the miRNAs profiles obtained after RNA extraction from 1×10^6^ or 3×10^6^ cells were compared using the same amount of total RNA for the RT step (130 ng). Experiments were also performed in duplicate and the mean Ct values of miRNAs detected in both cases was calculated. As expected, the quantity of RNA isolated from 3×10^6^ cells was higher than that obtained from 1×10^6^ cells (94 vs. 41 ng/μL respectively). RNA qualities were comparable as assessed by Agilent (nano 6000 and small RNA chips). Using an equal amount of RNA, the total number of detected miRNAs was higher after extraction from 3×10^6^ cells compared to 1×10^6^ cells (63 and 35 miRNAs, respectively, 33 miRNAs detected in both settings). A Bland-Altman plot of their Ct values showed that the miRNAs detected using RNA isolated from 3×10^6^ cells had lower Ct values, with a mean difference in Ct of −1.46 compared to 1×10^6^ cells extraction (Figure 5A), reminiscent of the results obtained with sera. We calculated the correlation between the difference in Ct values and the GC content (ρ = 0.42, p = 0.02, Figure 5B). As observed in the case of serum samples, miRNAs whose detection is sensitive to the quantity of the starting material used for MN extraction seemed to exhibit low GC content. Of note, no bias was observed in the GC content (Figure 5C, ρ = −0.08, p = 0.47) when different quantities of total RNA (300 and 100 ng) were compared for the RT step.

To investigate whether these observations were limited to the MN kit or whether RNA extraction with the Qiagen kit could introduce similar bias. Total RNAs were extracted from 1×10^6^ or 3×10^6^ PBMCs using the Qiagen miRNeasy mini kit and further profiled with TLDA cards. Similar to the results obtained after MN extraction, a Bland-Altman plot of the Ct values of the 61 miRNAs detected in both settings showed that the mean difference in Ct values of miRNAs detected in 3×10^6^ cells vs 1×10^6^ cells equaled −1 (Additional file 3: Figure S3A). This difference tends to correlate with the GC content (ρ = 0.29, p = 0.02, Additional file 3: Figure S3B although to a lesser extent, than observed in the case of MN extraction. Together these results confirmed at a genome-scale level and for two different miRNAs extraction kits previous observations made with Trizol RNA extraction procedures from human cell lines [13]. Kim *et al.* also reported that miRNAs extraction could be biased by RNA structures [13]. The existence of such bias was evaluated in our settings but any significant correlation was observed between the thermodynamic stability of miRNAs, as assessed by Quikfold, and the difference in Ct values (Additional file 4: Figure S4). Hence, this suggests that the extraction procedures used here did not introduce biases related to miRNA structures (Additional file 4: Figure S4).

## Discussion

Circulating miRNAs have recently emerged as non-invasive biomarkers of diverse pathologies. For a routine clinical use, technical standardization in the preparation of the samples and profiling methods are therefore important. The first factor that can affect the reproducibility of results is the quality of isolated RNA from human blood samples. In this study, we performed an independent cross-comparison of three extraction procedures for miRNA isolation from human blood samples using Macherey-Nagel, Qiagen, and Norgen kits. In order to facilitate comparison, we have deliberately chosen not to modify the manufacturer’s protocols. The originality of our study is that we compared miRNA profiles based on a TLDA platform.

The miRNAs were further profiled using the genome-scale Taqman Low Density Array technology. We indeed verified that this technology was reproducible in term of qPCR (ρ = 0.93) and RT (ρ = 0.90), with good agreement in Bland-Altman plots. Similarly, Chen et al*.* tested TLDA reproducibility on miRNAs detected in two different RTs and two different qPCRs using rodent cards and RNAs isolated from proliferating murine myoblast cells with Trizol method (from 500 ng RNA without pre-amplification and from 150 ng with or without pre-amplification) [20]. The comparison of the two replicates showed a strong correlation (ρ =0.978 for experiment using 500 ng RNA and ρ = 0.985 and ρ = 0.990 using 150 ng without and with pre-amplification, respectively). Wang and colleagues observed a correlation coefficient of 0.812 in the TLDA results using the same sample (human osterosarcoma xenografts, RNA isolated by Trizol, no pre-amplification) [21]. Jensen *et al.* tested specificity, reproducibility and sensibility of TLDA and miRCURY platforms using both synthetic miRNAs and plasma samples isolated by miRNeasy kit from Qiagen [22]. Concerning TLDA platform (protocol with pre-amplification) and plasma sample, reproducibility was assessed from one sample using two separate RT reactions and the products of each reaction were used in separate qPCR amplifications. The comparison of every duplicate pairs demonstrated a median correlation coefficient of 0.96 (cutoff Ct < 30) [22].

Given that, the 3 kits were first compared for their performances in extracting miRNAs from PBMCs in term of RNA quantity and quality. However, the new Macherey-Nagel kit was more efficient in extracting miRNAs from sera. Another advantage of this kit is that, unlike the Qiagen and Norgen extraction kits, it does not require the cumbersome phenol/chloroform step. Several previous reports have compared miRNAs extraction kits. Notably, Kroh *et al.* have tested variations on two extraction kits from plasma and serum samples: Ambion mirVana PARIS (with addition of an additional organic extraction step), and the Qiagen miRNeasy kit (with a modified protocol to use 10 volumes of Qiazol reagent per volume of plasma or serum). They showed that, although both protocols have proven effectiveness, the Qiagen protocol appears to produce 2–3-fold greater RNA yield [12]. Likewise, Li and colleagues evaluated the performances of the miRNeasy kit (Qiagen, CA), the miRVana PARIS kit (Ambion, TX) and the total RNA isolation kit (Norgen Biotek, Canada) [14]. They concluded that RNAs isolated by the Qiagen or Ambion kits had better quality (in terms of % of miRNAs in small RNA fraction) than those extracted with the Norgen kit. In term of RNA quantity, the concentrations of miRNAs in serum were 49 pg/μL, 29 pg/μL and 12 pg/μL from the Qiagen, Ambion and Norgen kits, respectively. Here, we obtained comparable amount of RNAs ranging from 11 (Norgen) to 80 (Macherey-Nagel) pg/μL.

Using TLDA profiling, we showed that the Macherey-Nagel kit allowed the detection of more miRNAs than the Qiagen kit (83 vs 41) in serum. Previous reports have shown a higher number of miRNAs detectable from serum samples by TLDA (around 170 miRNAs with RNA isolation with the Qiagen miRNeasy or the Ambion miRVana miRNA kits) [23–26]. However, in these studies, TLDA experiments were performed on serum pool of 10 to 20 samples.

One striking finding in our study is that, comparing two different volumes of serum or PBMCs numbers used to extract miRNAs with the Macherey-Nagel or the Qiagen kits, we showed that the quantity of the biological samples directly impacted the GC content of the miRNAs detected. These results are reminiscent of the results obtained by Kim *et al*., who showed that specific miRNAs can be lost during RNA extraction using TRIzol protocol (not with the Ambion miRVana miRNAs kit) depending on their GC content and their thermodynamic stability [13]. These results were obtained using cells from different density culture as starting RNA materials and miRNAs were detected by northern blotting. With these findings, Kim *et al*. hypothesized that small RNAs could require larger RNA carriers [13]. However, our results do not support this hypothesis as we found similar GC content bias in serum samples (Figure 4B) wherein no large RNAs was detected (Additional file 1: Figure S1B). We rather postulate that the presence of additional compounds (proteins and/or lipids that are associated with miRNAs, [27, 28] and whose quantity increase with starting material) can affect the nature of the miRNAs extracted. These compounds could further be lost during the RNA purification procedure implying that their concentrations would not show intrinsic linear relationship between cell input and total RNA. However, their presence in the initial steps of the purification could truly influence the GC composition of the purified RNAs. Together with that of Kim *et al*., our study support the use of identical quantities/volumes for starting materials to compare miRNA profiles.

## Conclusion

Overall, our results emphasize the importance of comparing miRNAs extraction protocols in order to standardize RNA isolation and to compare miRNAs profiles. In fact, numerous high-profile preclinical studies have already yielded conflicting data and outcomes due to differences in methodologies [29, 30]. There is therefore an urgent need of protocol standardization to enhance the future prospects of extracellular miRNAs in diagnosis, prognosis, and surveillance, even in therapeutic application. These types of study are all the more warranted, as the assays based on the extracellular miRNAs expression signatures prove useful as a noninvasive test to guide a physician’s clinical decision on comprehensive management of patients.

## Electronic supplementary material

Additional file 1: Figure S1: Comparison of the quality of total RNA isolated with three kits. A- Examples of Agilent nano 6000 and small RNA profiles obtained from RNA isolated from PBMCs samples. B- Examples of Agilent small RNA profiles obtained from RNA isolated from serum samples. Fluorescence intensity of RNA fractions at different sizes and associated gel electrophoresis. (PDF 490 KB)

Additional file 2: Figure S2: Bland-Altman analysis of miRNAs Ct values between the three RNA samples isolated by Macherey-Nagel (MN) and Qiagen extraction kits. A: From PBMCs samples (1×10^6^ cells). Only miRNAs with Ct < 32 were considered. B: From serum (300 μL) samples, no cut-off. (PDF 1 MB)

Additional file 3: Figure S3: Assessment of bias in RNA isolation from PBMCs samples using the Qiagen kit, comparison of extraction from 3×10^6^ and 1×10^6^ cells with the same amount of RNA for RT (110 ng). A- Bland-Altman analysis 3×10^6^ versus 1×10^6^ cells. B- Plot of the difference in Ct values of the two conditions (x-axis) and the GC content of miRNAs detected in these two settings (y-axis). The Pearson correlation coefficient is indicated. TLDA datas from biological duplicate. Analysis using mean CT values of common miRNAs. Only miRNAs with Ct < 32 were considered. (PDF 225 KB)

Additional file 4: Figure S4: Assessment of bias in RNA isolation from serum and PBMCs using the Macherey-Nagel (MN) kit: difference in Ct values of the two conditions in function of the thermodynamic stability of miRNAs. A- PBMCs: extraction from 3×10^6^ and 1×10^6^ cells but same amount of RNA for RT (130 ng). B- Serum: extraction from 600 versus 300 μL. TLDA datas from biological duplicate. Analysis using mean CT values of common miRNAs. Only miRNAs with Ct < 32 were considered for PBMCs. No Ct cut-off was applied for serum miRNAs. (PDF 46 KB)

## References

[1] Etheridge A, Lee I, Hood L, Galas D, Wang K (2011). Extracellular microRNA: A new source of biomarkers. Mutat Res.

[2] Becker N, Lockwood CM (2013). Pre-analytical variables in miRNA analysis. Clin Biochem.

[3] Witwer KW (2013). Data submission and quality in microarray-based microRNA profiling. Clin Chem.

[4] Pritchard CC, Cheng HH, Tewari M (2012). MicroRNA profiling: approaches and considerations. Nat Rev Genet.

[5] Wang K, Yuan Y, Cho JH, McClarty S, Baxter D, Galas DJ (2012). Comparing the MicroRNA Spectrum between Serum and Plasma. PLoS One.

[6] Cheng HH, Yi HS, Kim Y, Kroh EM, Chien JW, Eaton KD, Goodman MT, Tait JF, Tewari M, Pritchard CC (2013). Plasma processing conditions substantially influence circulating microrna biomarker levels. PLoS One.

[7] Blondal T, Nielsen SJ, Baker A, Andreasen D, Mouritzen P, Teilum MW, Dahlsveen IK (2013). Assessing sample and miRNA profile quality in serum and plasma or other biofluids. Methods.

[8] Kirschner MB, Kao SC, Edelman JJ, Armstrong NJ, Vallely MP, van Zandwijk N, Reid G (2011). Haemolysis during sample preparation alters microRNA content of plasma. PLoS One.

[9] Pritchard CC, Kroh E, Wood B, Arroyo JD, Dougherty KJ, Miyaji MM, Tait JF, Tewari M (2012). Blood cell origin of circulating microRNAs: a cautionary note for cancer biomarker studies. Cancer Prev Res (Phila).

[10] Kirschner MB, Edelman JJ, Kao SC, Vallely MP, van Zandwijk N, Reid G (2013). The Impact of Hemolysis on Cell-Free microRNA Biomarkers. Front Genet.

[11] Tzimagiorgis G, Michailidou EZ, Kritis A, Markopoulos AK, Kouidou S (2011). Recovering circulating extracellular or cell-free RNA from bodily fluids. Cancer Epidemiol.

[12] Kroh EM, Parkin RK, Mitchell PS, Tewari M (2010). Analysis of circulating microRNA biomarkers in plasma and serum using quantitative reverse transcription-PCR (qRT-PCR). Methods.

[13] Kim YK, Yeo J, Kim B, Ha M, Kim VN (2012). Short Structured RNAs with Low GC Content Are Selectively Lost during Extraction from a Small Number of Cells. Mol Cell.

[14] Li Y, Kowdley KV (2012). Method for microrna isolation from clinical serum samples. Anal Biochem.

[15] Eikmans M, Rekers NV, Anholts JD, Heidt S, Claas FH (2013). Blood cell mRNAs and microRNAs: optimized protocols for extraction and preservation. Blood.

[16] Debey-Pascher S, Chen J, Voss T, Staratschek-Jox A (2012). Blood-based miRNA preparation for noninvasive biomarker development. Methods Mol Biol.

[17] Burgos KL, Javaherian A, Bomprezzi R, Ghaffari L, Rhodes S, Courtright A, Tembe W, Kim S, Metpally R, Van Keuren-Jensen K (2013). Identification of extracellular miRNA in human cerebrospinal fluid by next-generation sequencing. RNA.

[18] Zuker M, Markham N (2006). Quikfold from the DINAMelt web server.

[19] Kirschner MB, van Zandwijk N, Reid G (2013). Cell-free microRNAs: potential biomarkers in need of standardized reporting. Front Genet.

[20] Chen Y, Gelfond JA, McManus LM, Shireman PK (2009). Reproducibility of quantitative RT-PCR array in miRNA expression profiling and comparison with microarray analysis. BMC Genomics.

[21] Wang B, Howel P, Bruheim S, Ju J, Owen LB, Fodstad O, Xi Y (2011). Systematic evaluation of three microRNA profiling platforms: microarray, beads array, and quantitative real-time PCR array. PLoS One.

[22] Jensen SG, Lamy P, Rasmussen MH, Ostenfeld MS, Dyrskjot L, Orntoft TF, Andersen CL (2011). Evaluation of two commercial global miRNA expression profiling platforms for detection of less abundant miRNAs. BMC Genomics.

[23] Cui L, Qi Y, Li H, Ge Y, Zhao K, Qi X, Guo X, Shi Z, Zhou M, Zhu B, Guo Y, Li J, Stratton CW, Tang YW, Wang H (2011). Serum microRNA expression profile distinguishes enterovirus 71 and coxsackievirus 16 infections in patients with hand-foot-and-mouth disease. PLoS One.

[24] Qi Y, Cui L, Ge Y, Shi Z, Zhao K, Guo X, Yang D, Yu H, Shan Y, Zhou M, Wang H, Lu Z (2012). Altered serum microRNAs as biomarkers for the early diagnosis of pulmonary tuberculosis infection. BMC Infect Dis.

[25] Song MY, Pan KF, Su HJ, Zhang L, Ma JL, Li JY, Yuasa Y, Kang D, Kim YS, You WC (2012). Identification of serum micrornas as novel non-invasive biomarkers for early detection of gastric cancer. PLoS One.

[26] Gui J, Tian Y, Wen X, Zhang W, Zhang P, Gao J, Run W, Tian L, Jia X, Gao Y (2011). Serum microRNA characterization identifies miR-885-5p as a potential marker for detecting liver pathologies. Clin Sci (Lond).

[27] Arroyo JD, Chevillet JR, Kroh EM, Ruf IK, Pritchard CC, Gibson DF, Mitchell PS, Bennett CF, Pogosova-Agadjanyan EL, Stirewalt DL, Tait JF, Tewari M (2011). Argonaute2 complexes carry a population of circulating microRNAs independent of vesicles in human plasma. Proc Natl Acad Sci U S A.

[28] Turchinovich A, Weiz L, Langheinz A, Burwinkel B (2011). Characterization of extracellular circulating microRNA. Nucleic Acids Res.

[29] Ioannidis JP (2005). Why most published research findings are false. PLoS Med.

[30] Ioannidis JP, Allison DB, Ball CA, Coulibaly I, Cui X, Culhane AC, Falchi M, Furlanello C, Game L, Jurman G, Mangion J, Mehta T, Nitzberg M, Page GP, Petretto E, van Noort V (2009). Repeatability of published microarray gene expression analyses. Nat Genet.

