# Supplementary material for: Comparison of different extraction techniques to profile microRNAs from human sera and peripheral blood mononuclear cells
Source: BMC Genomics. 2014 May 23;15(1):395. doi: 10.1186/1471-2164-15-395 (PMC4041998; doi:10.1186/1471-2164-15-395)
Supplement: Supplementary file 1 — Additional file 1: Figure S1: Comparison of the quality of total RNA isolated with three kits. A- Examples of Agilent nano 6000 and small RNA profiles obtained from RNA isolated from PBMCs samples. B- Examples of Agilent small RNA profiles obtained from RNA isolated from serum samples. Fluorescence intensity of RNA fractions at different sizes and associated gel electrophoresis. (PDF 490 KB) [file 12864_2013_6086_MOESM1_ESM.pdf]

A. PBMCs

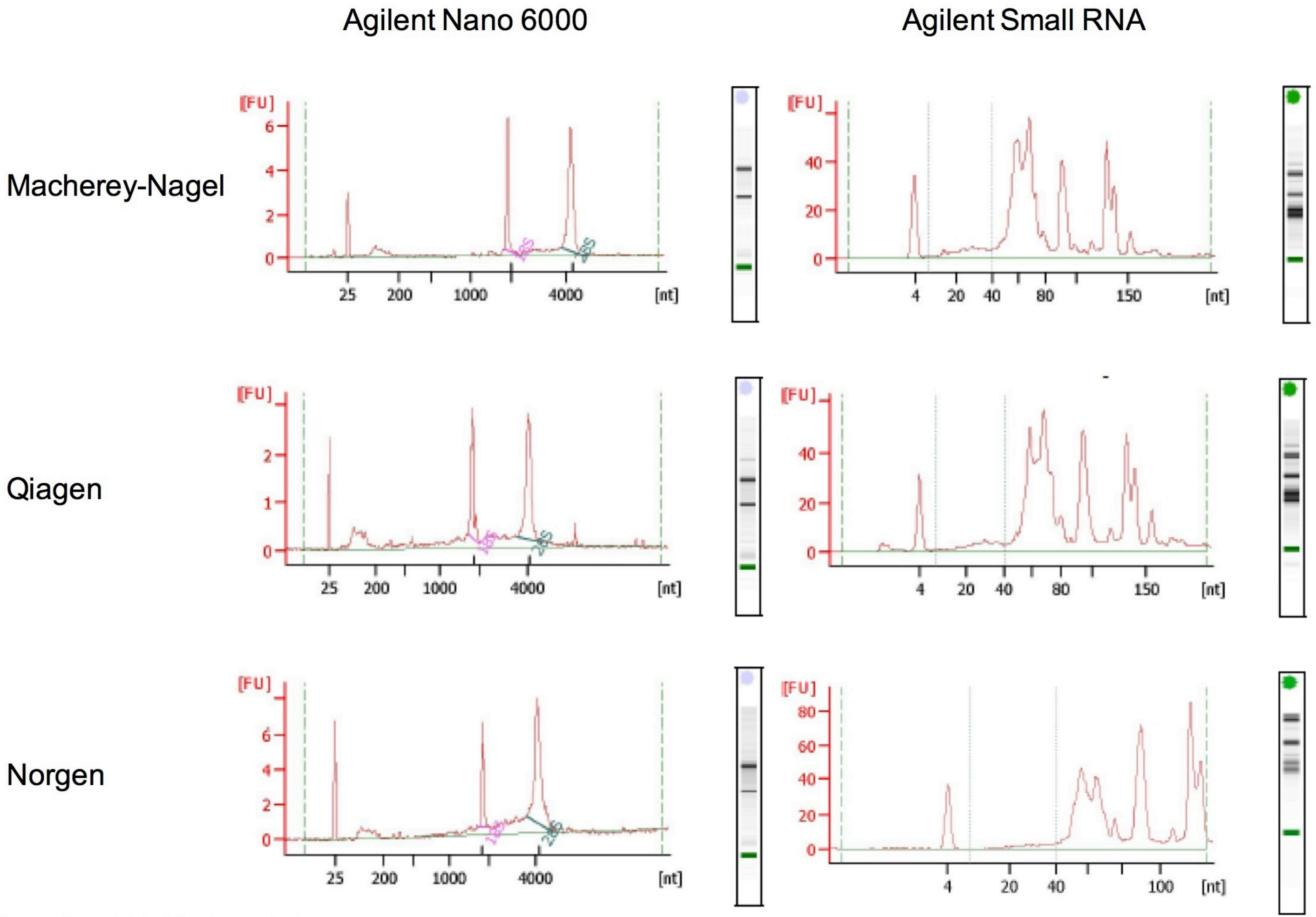

Figure Suppl 1A. Monleau et al.

B. Serum

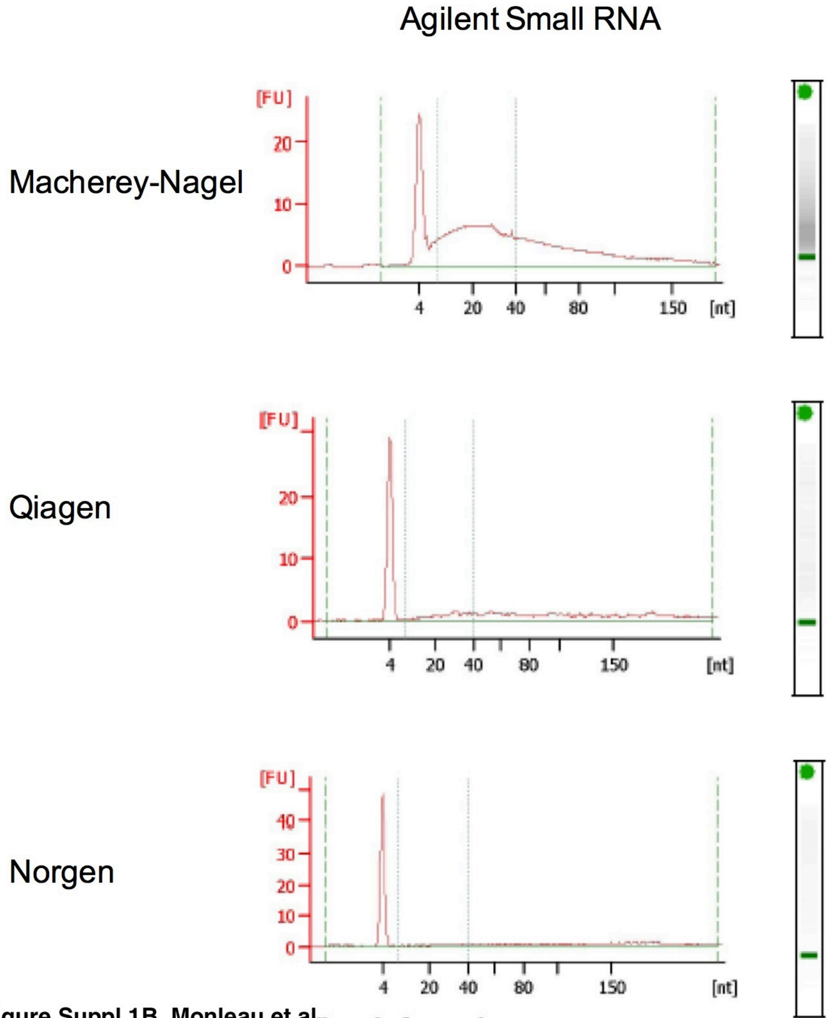

Figure Suppl 1B. Monleau et al.
