# Supplementary figures and images for: Comparison of different extraction techniques to profile microRNAs from human sera and peripheral blood mononuclear cells
Source: BMC Genomics. 2014 May 23;15(1):395. doi: 10.1186/1471-2164-15-395 (PMC4041998; doi:10.1186/1471-2164-15-395)

A. PBMCs

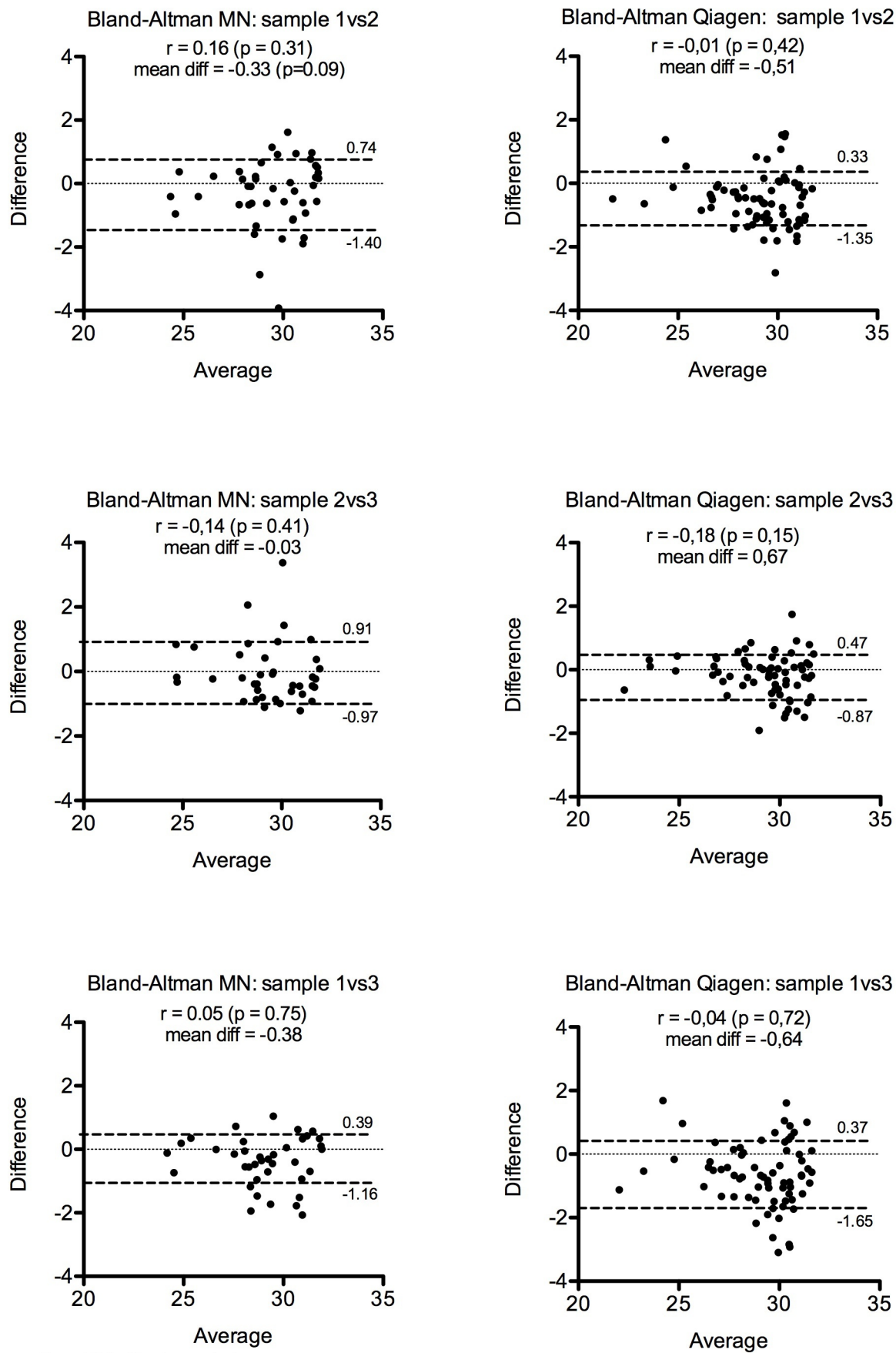

Figure Suppl 2A. Monleau et al.

B. Serum

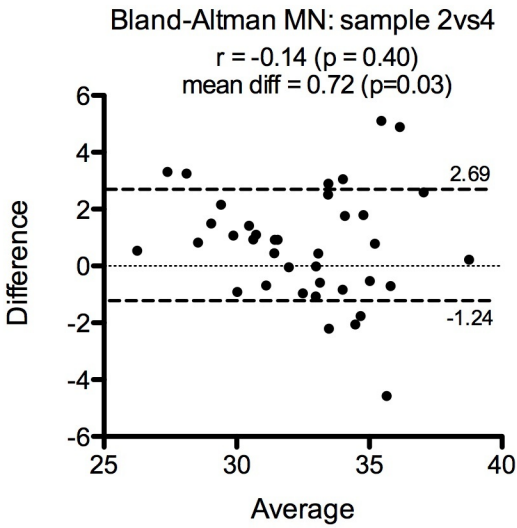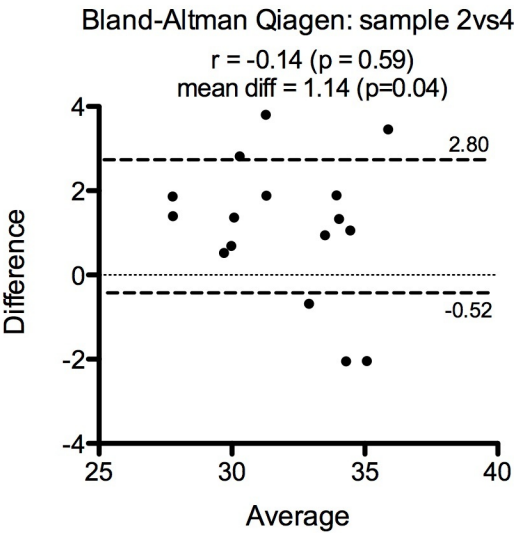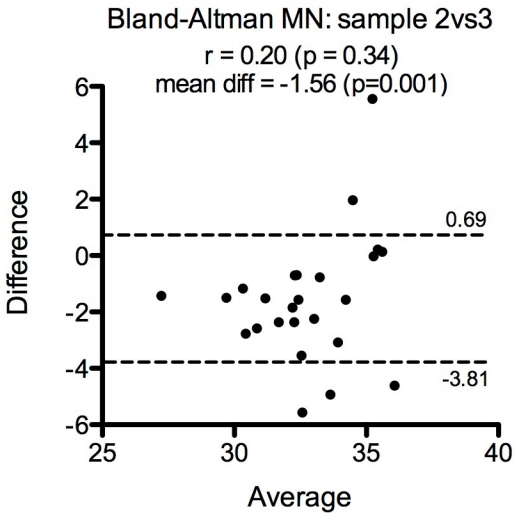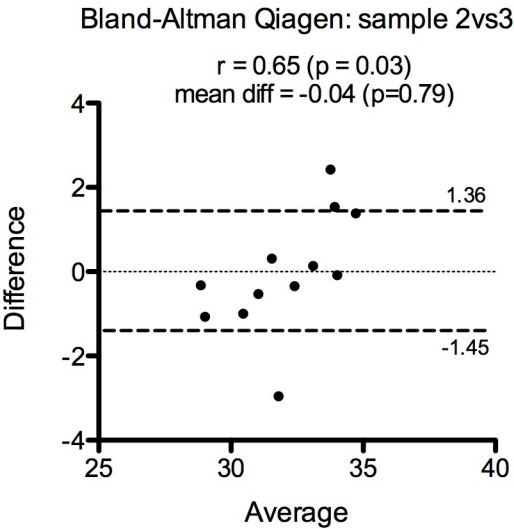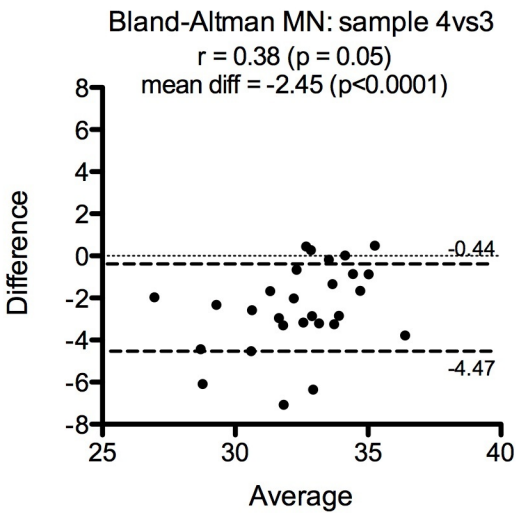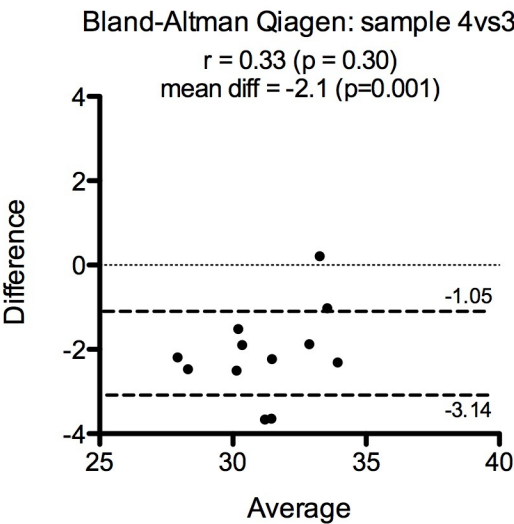

Figure Suppl 2B. Monleau et al.

Supplement: Supplementary file 2 — Additional file 2: Figure S2: Bland-Altman analysis of miRNAs Ct values between the three RNA samples isolated by Macherey-Nagel (MN) and Qiagen extraction kits. A: From PBMCs samples (1×10^6^ cells). Only miRNAs with Ct < 32 were considered. B: From serum (300 μL) samples, no cut-off. (PDF 1 MB) [file 12864_2013_6086_MOESM2_ESM.pdf]

**A. PBMCs**

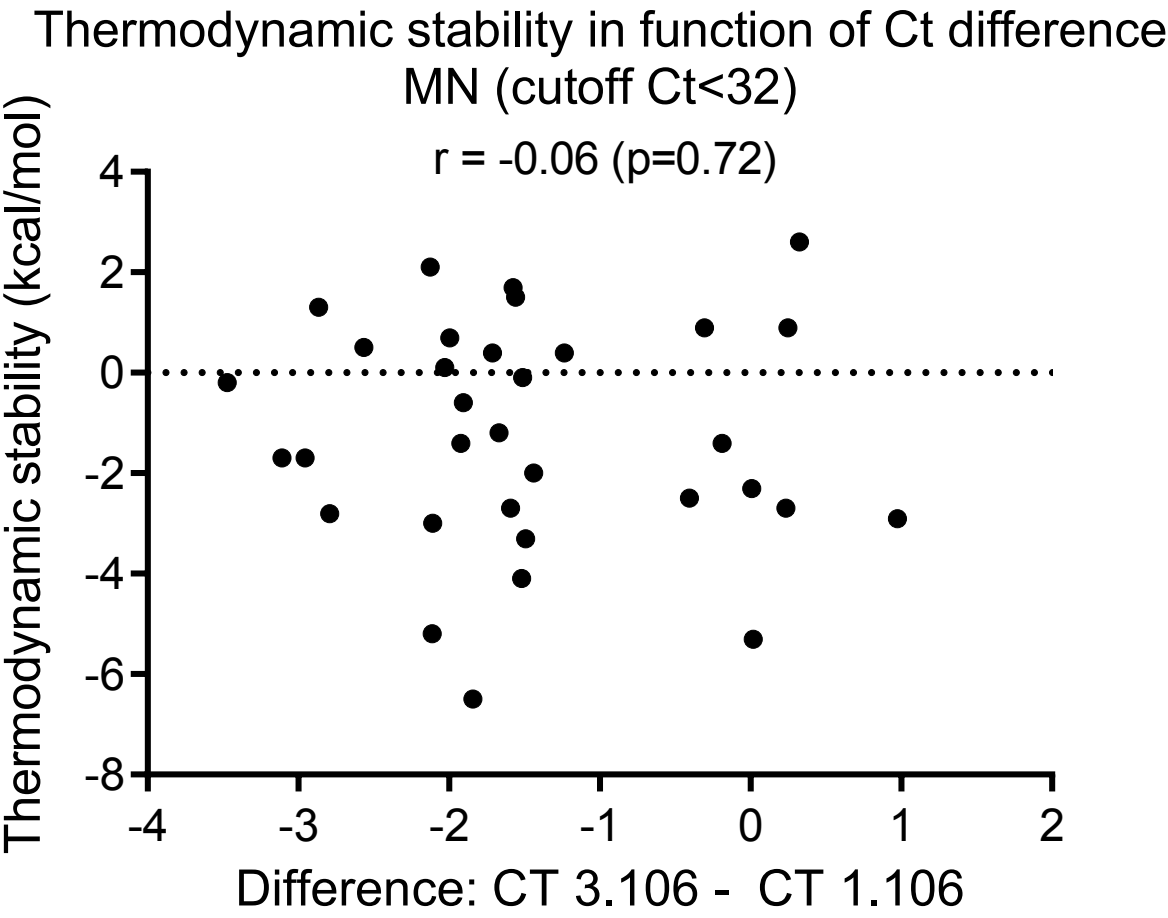

**B. Serum**

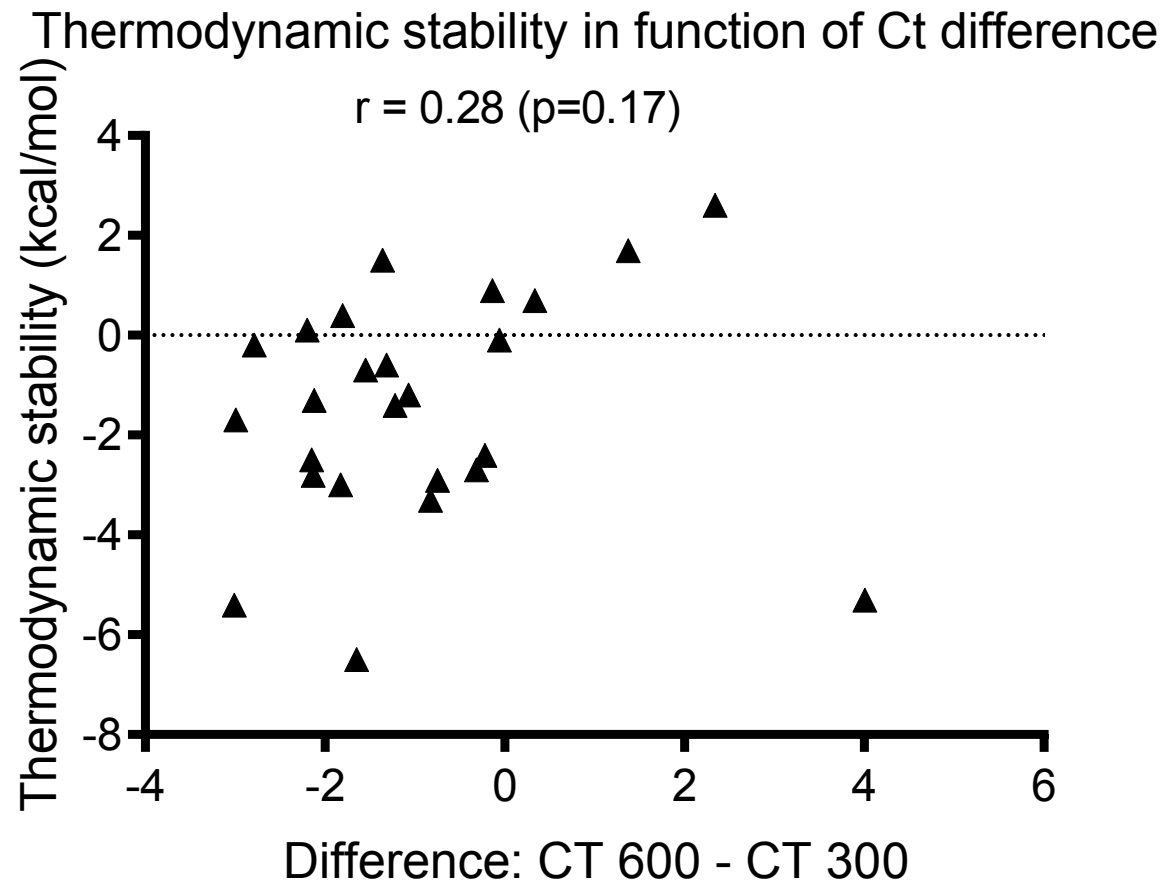

**Figure Suppl 4. Monleau et al.**

Supplement: Supplementary file 4 — Additional file 4: Figure S4: Assessment of bias in RNA isolation from serum and PBMCs using the Macherey-Nagel (MN) kit: difference in Ct values of the two conditions in function of the thermodynamic stability of miRNAs. A- PBMCs: extraction from 3×10^6^ and 1×10^6^ cells but same amount of RNA for RT (130 ng). B- Serum: extraction from 600 versus 300 μL. TLDA datas from biological duplicate. Analysis using mean CT values of common miRNAs. Only miRNAs with Ct < 32 were considered for PBMCs. No Ct cut-off was applied for serum miRNAs. (PDF 46 KB) [file 12864_2013_6086_MOESM4_ESM.pdf]
