# Supplementary material for: Comparison of different extraction techniques to profile microRNAs from human sera and peripheral blood mononuclear cells
Source: BMC Genomics. 2014 May 23;15(1):395. doi: 10.1186/1471-2164-15-395 (PMC4041998; doi:10.1186/1471-2164-15-395)
Supplement: Supplementary file 3 — Additional file 3: Figure S3: Assessment of bias in RNA isolation from PBMCs samples using the Qiagen kit, comparison of extraction from 3×10^6^ and 1×10^6^ cells with the same amount of RNA for RT (110 ng). A- Bland-Altman analysis 3×10^6^ versus 1×10^6^ cells. B- Plot of the difference in Ct values of the two conditions (x-axis) and the GC content of miRNAs detected in these two settings (y-axis). The Pearson correlation coefficient is indicated. TLDA datas from biological duplicate. Analysis using mean CT values of common miRNAs. Only miRNAs with Ct < 32 were considered. (PDF 225 KB) [file 12864_2013_6086_MOESM3_ESM.pdf]

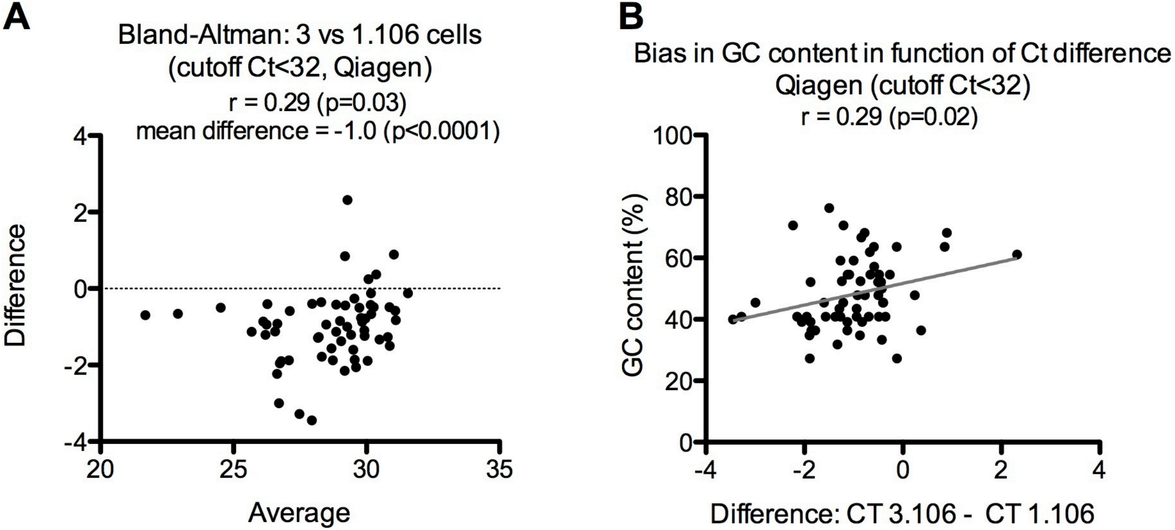

Figure Suppl 3. Monleau et al.
